# On the Hereditary Transmission of Pulmonary Tuberculosis

**Published:** 1867-05

**Authors:** A. P. Dutcher

**Affiliations:** Cleveland, O.


*On the Hereditary Transmission of Pulmonary Tuberculosis.* By Prof. A. P. Dutcher, M. D., of Cleveland, O.

There is no principle in medical science better established than the hereditary transmission of disease. Indeed it is an inexorable law of our being, and dates back to the fall of man, when the Almighty revealed to the race, that he would "visit the sins of the fathers upon the children to the third and fourth generation." Awful and impressive as this enunciation is, the warning is sometimes unheeded by the medical skeptic, and set at nought by those who are suffering its inflictions. Human nature is so debased; the influence of self-love so overwhelming and blinding to reason and judgment, that even while possessing numerous facts to guide them to the truth, thousands still resist the conviction, and suffer the penalty due to violated law.

It is true, as remarked by M. Renaudin, "that man appears to possess an independent existence, isolated from his birth from those who begat him, although there is but little apparent relation between his ripe age and first infancy; it is not the least true that behind the characters peculiar to his individuality, we can discover certain typical signs, some of which betray his nationality, and others relating to his family. These typical signs are to be encountered not only in his physical organization, but also found in his idiosyncrasies; and if tradition is of any force as regards manners and customs, inheritance is of great value as relates to the tastes and habits. It is in fact manifested in transmission from generation to generation of the most inveterate maladies, before which art is obliged to confess its weakness; and it is with difficulty prophylactics ward off the sad results."

### I. HEREDITARY INFLUENCE OF PHTHISIS.

I know of no disease in which we have so marked an exhibition of the doctrine of hereditary transmission as the one now under consideration. It is the outstanding type of all others. Whatever is characteristic of hereditary transmission in other maladies, finds its counterpart in the history of this. Peculiarities of mind, special configurations of body or features of countenance, are not more decidedly transmitted from parent to offspring, than the constitutional taint of pulmonary tuberculosis. It is not, in my judgment, simply the influence of a temperament, but a settled inherent predisposition to the deposit of tubercle in the lungs, and is propagated from one generation to another with more frequency than any other disease. And I am well satisfied, that when the mode of keeping medical statistics in this country shall become more uniform and perfect, they will show hereditary predisposition, in at least one-half of all who perish with the disease.

As a general thing, pulmonary tuberculosis is more frequently transmitted to the younger than the older children of a family, and more commonly to the females than the males. In a table compiled from the Bromptom Hospital Reports, London, I find that in one hundred cases of phthisis, the disease is transmitted by the father four times, and by the mother thirteen times. The reason for this may be found in the fact, that the females are more exposed to the same inducing causes as their maternal parent. I have known several families where the disease was confined exclusively to the females; the mother and daughters perishing with it, while the father and sons were exempt. We sometimes witness the same thing in cancer. I am acquainted with the history of a family, where for three generations, nearly every female died with the malady, while there was not an example among the males.

But we sometimes see children of a family perish with pulmonary tuberculosis, of which the parents exhibit no signs, when subsequently, the father or mother or both are attacked, and thus the departure of the disease, which exerts a kind of anticipatory action in the offspring, is disclosed. Several years since I attended two young men in a family that was supposed to be entirely free from phthisis; they died with very pronounced symptoms of the disease. Their mother at the time of their death appeared to be in vigorous health. Six months subsequently she fell a victim to the same malady, thus exhibiting the existence of an hereditary influence, the effect of which had preceded the manifestation. Again, on the other hand, we frequently see whole families of children cut off with phthisis, whose parents have shown no signs of the disease, living to old age, and perishing with other maladies. I have the history of a family of twelve children, all of whom perished with pulmonary tuberculosis but one; the mother died with dysentery a few years since, and the father is still living, over ninety years of age.

You must not, however, infer from anything that I have said, that pulmonary tuberculosis, when produced by hereditary transmissions, commences at birth; for I never met with but one case of congenital pulmonary tuberculosis since I commenced the practice of medicine, and this was in an infant of a prostitute, suffering from constitutional syphilis. The child died a few days after birth. It was supposed that she had poisoned it. But this was not verified by *post mortem.* Its little lungs were uniformly occupied with miliary tubercles, about the size of a pin's head, from their summit to their base, constituting one of the most perfect specimens of this variety of tubercular deposit that I have ever seen. In most instances you will find that phthisis pulmonalis is developed by growth, or some other circumstance in life. A parent, for example, has it in middle life; his son does not get it until about the same period,—sooner or later. In this way the disease may remain latent for years before it is manifested. It has, however, been observed, that under the influence of hereditary predisposition, the disease manifests itself at an earlier age than at which it is ordinarily developed independent of other causes.

Another interesting fact connected with the hereditary transmission of the tubercular predisposition is, that it may sleep through one generation only to awake in the next with redoubled energy. Dr. L. M. Lawson says "Every practitioner has met with numerous examples in which both parents were apparently free from taint, while their offspring suffered from tubercular or scrofulous affections; but on pushing the inquiry further, it would be found that uncles, aunts, or grandparents, had suffered from similar diseases. A young man, laboring under precursory signs of phthisis, presented himself to me for treatment; and the history of the case revealed the fact that he had lost four sisters and two brothers with consumption, and he, the remaining child, was now threatened. His father died at the age of forty-five, without any signs of pulmonary difficulty; his mother, aged fifty-five is living, in the enjoyment of good health. On further inquiry, I learned that the grandfather on the mother's side was said to have died of consumption of the bowels; and also, that his mother and sister died of consumption, and several of his brother's children perished in a similar manner. This is a very remarkable case. The maternal grandfather has some form of scrofulous or tubercular disease; but the daughter (mother of the patient) fifty-five years of age, and yet her *seven* children become the subjects of consumption; six die, and the seventh manifests decided symptoms of the approach of the disease."

Again, we sometimes see individuals marry when actually suffering under the first stage of phthisis; and we have often known a single year to circumscribe the existence of one of the parties, and occasionally both. Not unfrequently an offspring is the result of this union, who is almost sure to fall a victim to some form of tubercular disease. Infants, as we have already remarked, very seldom die with pulmonary tuberculosis; the taint commonly manifests itself in the brain or bowels. In the earlier part of my practice, I had under my care a young man who gave every evidence of incipient phthisis pulmonalis. He inherited a predisposition to the disease from his mother. At a period of temporary improvement in health, he married a young woman of good constitution, having no proclivities to the disorder

He fell a victim to it about five years afterwards. The results of this union were three children, all of whom perished when they were about a year old from tubercular meningitis. His wife subsequently married a man free from all tubercular taint: they had four children; and so far as I know, not one of them ever manifested a single symptom of tubercular disease; and I was the family physician for more than fifteen years.

But children so frequently perish with tubercular disease, whose parents never have exhibited any traces of the malady, that some medical skeptics like Dr. Walshe, of London, ignore any hereditary influence in the case. I have no sympathy with such teachers. I have not the least hesitation in saying, that there are few cases of turbercular disease in children, which cannot be traced directly to the parent as the source of its origin. It is true, that in every instance the parent may not labor under the tubercular diathesis, but he may suffer from other constitutional maladies, which are known to produce tubercular disease in children. How often do we see the offspring of those who have constitutional syphilis die with the most aggravated forms of tuberculosis? What vast numbers of children perish with tubercular meningitis whose parents are inebriates? All going to prove that the sins of the fathers are visited upon the children. Who will deny that even the secret vices and excesses of early youth, may not be attended by scrofula and phthisis in offspring? The mental and physical condition of the parent, at the time of conception, has a most powerful influence for good or evil upon the future destinies of mankind. Shakspeare appears to have a vivid idea of this, when he put the following in the mouth of Edmund, in King Lear:

> " Why brand they us
> With base? with baseness? bastardy? base, base?
> Who, in the lusty stealth of nature, take
> More composition and fierce quality,
> Than doth, within dull, stale, tired bed,
> Go to the creating a whole tribe of fops,
> Got 'tween asleep and wake?"

If mankind were not begotten in the manner spoken of by the great dramatist, I most firmly believe he never would present himself before us in the degenerated physical and mental condition that he does; his nervous system would never be so early and irregularly developed, as to make his subsequent life a curse to himself, and full often present him to our view, a driveling idiot, the wretched victim of insanity or of tuberculosis.

II. HOW TO PREVENT THE TRANSMISSION OF PHTHISIS.

It is the settled conviction of some of our best medical writers, who have expressed an opinion on this subject, that the hereditary predisposition to tubercular disease can in a great degree be prevented by attention to the laws of health and matrimonial alliances of successive generations. "If," says Sir James Clarke, in his elaborate work on consumption, "a more healthy and natural mode of living were adopted by persons in that rank of life, which gives them the power of choice, and if more consideration were bestown on matrimonial alliances, the disease which is so often entailed on their offsprings might not only be prevented, but even the predisposition to it extinguished in those families, in the course of a few generations."

The propriety of avoiding intermarriages with those families who give evidence of being tainted with the disorder, will not be questioned by any who has made the subject of phthisis a particular study. I think we should boldly protest against every union which will have the slightest tendency to entail on posterity this fatal and dreadful disease—God and humanity require it. The physician is the guardian of the public health. His mission is to prevent as well as cure diseases. It is with the living, moving, present race he has to do; with a being who contains within himself the germ of the highest mental and corporeal excellence. Alas! that the web of depravity and ignorance, that has so assiduously been wound around him, even from his earliest existence, should have so long opposed his physical and moral regeneration.

I have often felt in my intercourse with mankind, that it was almost a fruitless task to advise the practice of reason and common sense to those who were about to enter into the matrimonial state, especially to those who believe that love is invincible and uncontrollable; yet I have occasionally seen it attended with good. We know that all our passions are apt to take on morbid action by over excitement. This is especially the case with love, as it relates to the sexes. When an individual is thus affected, there is a peculiar overpowering influence that takes possession of the mind, which is fruitful in sighs, tears, and sleepless nights, caused by a pretty foot, a keen eye, a winning smile, or a tender expression; and one thus affected deems himself most desperately and irrecoverably in love. But, unless the being after whom he sighs happens to possess some of the standard excellences of character, he will perhaps find, when too late, that he has entered upon a course from whence there is no retraction. How often is it the case, that those who have been once as blind as the little god himself, are at length aroused from their sweet dreams of fancied bliss to the sad realities of wedded unhappiness.

But he is not often the only sufferer; others reap the fruit of his errors; posterity have a greater interest at stake than is often supposed, and which is still less oftener consulted. Suppose a couple, both the branches of a stock affected with tubercular disease, fall desperately in love, and there can be no objection to their union in respect to the moral worth of either party, is marriage, with their predispositions to the disease, justifiable or expedient? Or, in other words, will they be excusable for knowingly entailing such a fatal malady upon posterity? Are they excusable for perpetuating a disease that blights the fairest prospects of the race, and consigns so many to a premature grave? It would be better for them to suffer in their feelings, than that a numerous progeny should endure the ills of this wasting disease. Such reflectious and sentiments enunciated by the scientific and upright physician, enforced by the spirit of truth, must touch the very heart; they cannot fail to reach the conscience. Reason would follow the dictates of conscience; but feeling, passion, and self-love prompts to a violation of moral and organic law.

I am well satisfied, from my own personal observation, that there is no relation in life which contributes more to the happiness or health of mankind, when judiciously formed, than matrimony; and yet, strange to say, we frequently see individuals enter into it with as little reason as if they were incapable of it. Passion rules the hour, and when blinded and maddened by its influence, they hurriedly enter into this important relation; and it is a truth which cannot be denied, that very many of these marriages formed in haste, when the parties are intoxicated with passion, are insensible to everything but its influence, end in mutual coldness, disgust, and faithlessness to the marriage vows.

It is true, a couple for a time may live on little less than love; but if there is a great inequality in temper, disposition, or education; or if the habits of living of one or both have been much more expensive than their means will warrant in the new relation they are about to form, they may well ponder the step they are about to take. Marriage alone does not confer happiness; but when formed with due reflection and proper principles, it will result in prosperity, and be followed by the most enduring affection.

### III. THE MEDICAL MANAGEMENT OF INDIVIDUALS WHO HAVE A HEREDITARY PREDISPOSITION TO PHTHISIS.

Under the term *medical management* we include both hygienic and therapeutical measures. As we cannot prevent phthisical individuals from getting married and having children, we will often be called upon to give instructions as to the best mode of rearing them. Those who are wise will be guided by our advice; those who are not will neglect it, and as a consequence, see their offspring fill a premature grave.

In this case medical management cannot commence too soon. It should be begun in early infancy where the slightest tubercular taint is manifested in the parent, and there is well grounded fear that it inherits the same habit. The health of the mother during the time of nursing is a matter of great importance; everything to promote it should be rigidly insisted upon. The means to accomplish this have already been pointed out in another lecture.

The greatest care should be taken at all times that the child is provided with sufficient nutriment easy of digestion, excessive repletion being carefully avoided. The health of the digestive organs must be faithfully watched, and everything that disagrees with them strictly prohibited. The apartments in which it is kept should be well ventilated and of a moderate temperature; extremes of temperature should at all times be avoided.

When the weather permits, it may be daily exposed to the outer air; bathing and all the other means of hygiene should be attended to as the nature and circumstances of the case may demand. When the child arrives at that age when it is capable of taking exercise, it should be encouraged to engage in active sports, such as jumping, playing ball, and the like; but excessive indulgence should be avoided. The training of the mind should also keep pace with the body; but in no instance should it interfere with a full share of bodily exercise.

As puberty approaches, the greatest watchfulness should be had, that during this interesting period no bad habits be acquired—especially solitary vices, which expose the system to various derangements of health and diseases of a troublesome and fatal character. Walking and riding on horseback or light gymnastics, and the use of the tepid or cool bath, as personal experience may indicate, are now to be regularly and systematically practised. There are few things which contribute so much to the health and vigor of the human body as a clean skin. Few persons have any idea of the vast amount of effete matter that is constantly eliminated through its pores; when these are continually obstructed the system can never be in perfect health. See to it then, that it is thoroughly cleansed every way.

The exterior of the body should also be well protected from vicissitudes of temperature by suitable clothing. Flannel beyond all question is the best material for this purpose, and in a climate like ours, where it does not positively disagree with the skin, it should be worn by every one who is in the least predisposed to phthisis. Keep the exterior of the body clean and warm, and there will not be much danger of internal inflammations and fatal congestions. I am well satisfied that if more attention was paid to the clothing of children, among even the wealthiest class of the community, who have it in their power to dress their offspring as they please, the mortality of the race would be very much lessened. Croup, broncho-pneumonia, and some other diseases which so frequently demand the aid of the physician, would be exceedingly rare. But so long as society continues to be governed by the frivolities of fashion in the matter of dress, we cannot expect parents to be governed by the laws of nature or reason on this subject. Hence the necessity in this instance of enforcing our advice with special emphasis. * * * * * *

[*Med. and Surg. Rep.*

